# Consumption Habits and Perception of Plant-Based Milk and Dairy Alternatives Among Vegetarians and Omnivores: A Case Study of Consumers in Slovenia

**DOI:** 10.3390/foods15050961

**Published:** 2026-03-09

**Authors:** Kaja Kranjc, Andreja Čanžek Majhenič, Tanja Pajk Žontar

**Affiliations:** 1 University of Ljubljana, Biotechnical Faculty, Department of Food Science and Technology, Jamnikarjeva 101, SI-1000 Ljubljana, Slovenia; kaja.kranjc@bf.uni-lj.si; 2 University of Ljubljana, Biotechnical Faculty, Department of Animal Science, Groblje 3, SI-1230 Domžale, Slovenia; andreja.canzek@bf.uni-lj.si

**Keywords:** plant-based milk and dairy alternatives, consumer perception, dietary patterns, vegetarian, vegan, dairy, socio-demographic determinants, nutrition labelling, Slovenia

## Abstract

Background: Plant-based milk and dairy alternatives (PBMDAs) are increasingly consumed in Europe, yet evidence from Central Europe remains limited. This study investigated PBMDA consumption habits and perceptions among adults in Slovenia. Methods: A cross-sectional online survey was conducted in June 2024 using a nationally stratified consumer panel (N = 1500). The questionnaire assessed socio-demographics, lifestyle, selected self-reported psychological indicators, dietary pattern, PBMDAs-related beliefs, and interpretation of nutrition and ingredient information. Descriptive statistics and chi-square tests were complemented by multinomial logistic regression and Bayesian analyses. Results: Most participants followed an omnivorous diet, while vegetarians and vegans constituted a minority. Compared with omnivores, vegetarians expressed more favorable perceptions of PBMDAs (health, sustainability, safety), whereas omnivores expressed greater trust in dairy’s nutritional adequacy and stronger concerns about processing and additives. PBMDA perceptions varied by socio-demographics: younger participants and women expressed positive views, and vegetarian/vegan diets were more common among women and higher-educated consumers. Vegetarians/vegans reported more anxiety and body dysmorphic concerns than omnivores. When nutrition information was anonymised, both groups tended to rate dairy as healthier, indicating persistent biases in product evaluation. Conclusions: PBMDA perceptions in Slovenia are strongly segmented by dietary pattern and socio-demographics, supporting the need for clearer nutrition communication.

## 1. Introduction

Over the past decade, the plant-based milk and dairy alternatives (PBMDAs) market in the European Union (EU) has grown steadily and is projected to continue expanding between 2024 and 2029. Forecasts predict an increase of approximately USD 2.6 billion over this period—a 76.47% rise—reaching USD 6 billion by 2029 [1]. In 2023, 28% of participants from selected European countries reported consuming PBMDAs at least once a week, reflecting a shift driven by consumers’ perception of such products as being healthier and environmentally friendlier than milk and dairy products [2,3,4], although this assumption is not always supported by evidence [4,5]. This sustained growth underscores the increasing acceptance and demand for PBMDAs across the EU [1].

Because consumers often rely on labels and category cues when evaluating PBMDAs, it is also relevant to consider the EU regulatory context for how these products are presented. Under Regulation (EU) No 1169/2011 [6], prepacked foods must carry a standardised nutrition declaration and ingredient list, and food names must not mislead consumers. In addition, protected dairy designations (e.g., “milk”, “cheese”, “yoghurt”) are reserved for products derived exclusively from milk under Regulation (EU) No 1308/2013 [7], meaning fully plant-based products cannot legally be marketed using these dairy terms and typically require clear descriptive names that distinguish them from dairy products. Finally, nutrition and health claims are regulated under Regulation (EC) No 1924/2006 [8], and voluntary vitamin/mineral fortification is governed by Regulation (EC) No 1925/2006 [9]—both directly relevant when consumers compare fortified PBMDAs with dairy.

PBMDAs can be classified into five main categories according to their primary ingredient: cereals (e.g., oats, rice), legumes (e.g., soy, peas), vegetables (e.g., potato), seeds (e.g., flaxseed, hempseed), and nuts (e.g., almond, cashew, coconut) [10]. Their nutritional profile varies considerably depending on the ingredients, formulation, and processing method [11,12,13]. Compared with milk and dairy products—rich sources of high-quality protein, calcium, phosphorus, and other micronutrients—PBMDAs generally contain less or even no protein and lower amounts of certain vitamins and minerals unless fortified [12,13,14,15,16,17,18]. Moreover, processing methods aimed at replicating the sensory qualities of dairy can result in products classed as ultra-processed, with higher levels of added sugars, saturated fats, and/or sodium [5,14,15,16,17,18,19].

Milk is a nutrient-rich secretion produced by lactating mammals to nourish offspring [20] and has long been processed into stable dairy products with distinct sensory properties (e.g., yoghurt, cheese, sour cream) and varying macronutrient profiles [21,22]. Bovine milk is typically ~85–87% water and ~13–15% dry matter, containing ~3.8–5.5% fat, ~2.9–3.5% protein, ~5% carbohydrates, and ~0.7% minerals [23]. Dairy proteins are highly digestible and provide all essential amino acids, and dairy is an important source of minerals such as calcium, phosphorus, and magnesium [21]. Compared with bovine milk, PBMDAs generally contain less energy and protein, while their micronutrient profile depends largely on the degree of fortification [5,17,23,24,25].

Consumer choices regarding PBMDAs are influenced by socio-demographic characteristics, with younger, female, and more highly educated individuals reporting higher willingness to replace dairy products with PBMDAs [26,27,28,29,30]. In addition, health beliefs, environmental concerns, ethical considerations, and sensory expectations are also important motives for adopting plant-based diets, with perceptions of environmental benefits, healthiness, and sustainability positively associated with PBMDAs consumption [26]. Across Europe, taste, perceived health benefits, and curiosity are the main drivers for trying PBMDAs, while affordability and availability affect regular consumption [15,16,17]. PBMDAs are often associated with a lower environmental footprint, while health issues such as lactose intolerance or dairy protein allergies are more delicate topics for some individuals [18,19]. At the same time, consumers frequently rely on simplified health cues—such as “plant-based” or “natural”—when evaluating these products, which may not always reflect their actual nutritional quality [4,26]. However, awareness of differences in protein content, micronutrient composition, and fortification practices is limited, contributing to nutritional misperceptions. Sensory characteristics of PBMDAs—especially flavour, texture, and mouthfeel—play a decisive role in repeat purchases, with products closely resembling dairy gaining wider acceptance among both vegetarians and omnivores [27,31]. Advances in food technology, including the use of emulsifiers, stabilisers, flavouring systems, and protein engineering, have substantially improved the sensory resemblance of PBMDAs to conventional dairy products, reducing perceptible differences in blind sensory evaluations [19,32].

In addition to socio-demographic factors, our study also considers the potential influence of mental health conditions and body image concerns on dietary decision-making and interest in PBMDAs. This is particularly relevant because psychological factors such as anxiety, depression, emotional regulation, and body dissatisfaction have been shown to impact dietary patterns and food choice behaviors [33,34,35,36,37,38,39,40,41,42,43,44]. Emerging evidence further suggests that dietary identities, including vegetarian and vegan patterns, may intersect with psychological characteristics in complex and bidirectional ways. Health belief models, such as those proposed by Rosenstock (1974) [44], suggest that perceptions of health risks and benefits significantly influence dietary decisions. These models are consistent with the notion that perceived health benefits can shape consumer attitudes toward food and influence food choices beyond purely nutritional considerations. Within this framework, individuals who perceive dairy products as contributing to weight gain, inflammation, or other adverse health outcomes may be more inclined to substitute them with PBMDAs perceived as healthier alternatives. Individuals with heightened anxiety or concerns about their body image often adopt restrictive eating behaviors, which may include choosing foods that are perceived as “clean,” “healthy,” or “controlled”, characteristics commonly associated with PBMDAs. For example, Zuromski et al. (2015) [35] found that women with eating pathology are more likely to follow vegetarian diets, which are often perceived as more “controlled” or healthier. Additionally, Saintila et al. (2024) [33] found that depression and anxiety are associated with emotional eating and restrictive eating behaviors, which can further influence dietary preferences. Similarly, Bardone-Cone et al. (2012) [36] and Timko et al. (2012) [38] reported that vegetarianism may, in some cases, coexist with elevated dietary restraint and eating disorder symptomatology, although motivations for adopting such diets vary substantially across individuals.

The relationship between anxiety and/or depression and dietary patterns is also emphasized in the literature. Matta et al. (2018) [34] observed that depressive symptoms were associated with vegetarian dietary patterns in a large cohort, although the directionality of this relationship remains unclear. On the other hand, several studies suggest that well-planned vegetarian and vegan diets are not inherently associated with higher levels of anxiety or depression once socio-demographic and lifestyle confounders are considered [40,41,42]. Other studies even suggest that vegetarianism may be associated with lower odds of depressive symptoms and better mental health outcomes in specific populations [43,44]. Importantly, these findings highlight that observed associations may reflect pre-existing personality traits, greater emotional sensitivity, or heightened health consciousness among individuals who choose plant-based diets, rather than causal effects of the diets themselves.

In line with this interpretation, recent research has highlighted that inconsistencies across studies may partly stem from heterogeneity within plant-based dietary groups and differences in underlying motivations. Taken together, these findings suggest that psychological characteristics may interact with dietary motivations in shaping interest in PBMDAs, but current evidence does not support a simple or unidirectional relationship between plant-based eating patterns and adverse mental health outcomes.

Despite the growing body of literature examining the nutritional quality and market development of PBMDAs, no study to this day in Slovenia has yet explored how consumption patterns and perceptions differ across consumer groups with distinct dietary orientations. In Slovenia, where traditional dietary habits remain deeply rooted but are gradually shifting to mirror broader European trends towards plant-based diets, understanding the motivations, preferences, and perceived barriers among both vegetarians and omnivores is particularly relevant for informing nutrition policy, public health recommendations, and product innovation.

This study, therefore, aims to investigate consumption habits and perceptions of PBMDAs among Slovenian vegetarians and omnivores, focusing on differences in frequency of consumption, ingredient preferences, perceived health benefits, and sensory evaluation. In particular, we investigated whether (i) lifestyle and body self-image are associated with age, sex, education level and income; (ii) dietary pattern (omnivorous vs. vegetarian) depends on socio-demographic background; (iii) dietary pattern is related to lifestyle characteristics (e.g., physical activity, smoking, healthy eating and mental health status); (iv) socio-demographic factors and dietary patterns influence consumers’ perceptions of PBMDAs and their motivations to replace conventional dairy products; and (v) dietary pattern affects consumer understanding and interpretation of nutrition and ingredient information on food labels.

By examining these factors, the research seeks to provide insights into how dietary orientation influences PBMDA acceptance and to contribute to strategies for promoting nutritionally balanced and sustainable food choices.

## 2. Materials and Methods

### 2.1. Study Design and Participants

This cross-sectional survey was conducted to assess consumption habits and perceptions of milk, dairy products, and PBMDAs among adults in Slovenia, with a focus on differences between vegetarians and omnivores. The study also examined the influence of socio-demographic factors on lifestyle, dietary patterns, and attitudes toward PBMDAs, as well as the potential role of self-consciousness about body image and the presence of body dysmorphic concerns in shaping dietary choices, including veganism.

Data were collected in June 2024 through an online questionnaire administered by an external market research agency, which maintains a large, nationally representative panel of Slovenian residents. The target population included individuals aged ≥18 years residing in Slovenia. Recruitment was carried out by sending invitations to panel members via email, with sampling stratified by age, sex, and geographic region to ensure representativeness.

After applying the inclusion criteria (Slovenian residency, age ≥18 years, and complete responses to all mandatory items), 1500 participants were included in the final dataset.

Ethical approval for the study was obtained from the Nutritional Research Ethnics Committee, Biotechnical Faculty, University of Ljubljana (KEP-4-3/2024).

### 2.2. Questionnaire Development

The questionnaire was developed based on a review of relevant scientific literature in the fields of nutrition and consumer behaviour. It comprised 28 questions organised into thematic sections. The first ten questions addressed socio-demographic characteristics, lifestyle-related behaviours, and mental health status, whereas the remaining items focused on dietary habits related to milk and dairy products, experience with PBMDAs, and attitudes towards such products. Both closed- and open-ended formats were used to allow for quantification and additional clarification of responses.

Prior to finalisation, the draft questionnaire was evaluated in a pilot test with 30 adult respondents to assess clarity, relevance, and overall structure. Minor revisions were made in response to the feedback received. The pilot test indicated an average completion time of approximately 15 min.

### 2.3. Data Collection Procedure

The survey was implemented online via the market research agency’s secure platform. Panel members received an email invitation containing a personalised survey link, which prevented multiple submissions from the same individual. Participants could complete the questionnaire using a desktop computer, tablet, or smartphone.

The system ensured completion of mandatory questions before progressing to the next section, and responses could not be changed once submitted (“forward-only” design). Quality control measures included automated screening for inconsistent responses and exclusion of incomplete submissions. Data were collected over a one-month period (June 2024), after which the anonymised dataset was provided to the research team for analysis.

### 2.4. Statistical Analysis

All statistical analyses were performed using R version 4.3.1 (R Foundation for Statistical Computing, Vienna, Austria). Descriptive statistics (frequencies and percentages for categorical variables; mean, standard deviation, median, and interquartile range for continuous variables) were used to summarise the characteristics of the study population. Group comparisons were conducted with the chi-square test, and effect sizes were expressed using Cramer’s V. Distribution of responses was visualised using bar charts. Both frequentist and Bayesian approaches were employed for complementary purposes. Chi-square tests with Cramer’s V provided widely used effect size measures for initial group comparisons. Bayesian multinomial logistic regression was additionally performed because it handles small cell sizes more robustly, provides direct probability statements about parameters, and allows incorporation of prior uncertainty—particularly relevant given the small vegetarian/vegan subgroup in our sample.

For multivariate analysis, a Bayesian multinomial logistic regression model was fitted using the brms package (version 2.21.0). The model estimated the probabilities of each outcome category while accounting for random effects. Convergence of the Markov chains was assessed using Rhat values (target value ≈ 1), and the effective sample size (ESS) was evaluated to ensure the reliability of parameter estimates. Results are presented as odds ratios (ORs) with corresponding 95% credible intervals (CrIs). A two-sided *p*-value < 0.05 was considered statistically significant.

## 3. Results

### 3.1. Socio-Demographic Characteristics and Lifestyle Indicators

The first part of the questionnaire was designed to obtain comprehensive information on socio-demographic characteristics (Table 1) and lifestyle-related behaviours with the aim of exploring whether these factors are associated with body self-image. Specifically, respondents were asked to report their sex, age, education level, marital status, type of residential area, weight (kg), and height (cm), from which body mass index (BMI, kg/m^2^) was calculated.

In addition, several items assessed lifestyle indicators, including physical activity, smoking, and alcohol consumption, as well as body satisfaction, the presence of self-reported disordered eating tendencies (e.g., body dysmorphic concerns, experiences with anorexia nervosa, orthorexia nervosa, or bulimia nervosa), and self-reported mental health conditions (e.g., anxiety, depression, or other mental health conditions). This section was structured to research how lifestyle and body self-image are associated with socio-demographic determinants such as age, sex, education, and income. Participants were able to choose multiple answers that applied to them. Results of self-reported lifestyle and body image characteristics of participants are presented in Table 2.

To examine the association between socio-demographic characteristics and lifestyle and self-reported body-image indicators, a Bayesian multinomial logistic regression model was estimated using sex, age, and marital status as predictors. Results are shown in Table 3.

Female respondents were significantly more likely to report dissatisfaction with their physical appearance (OR = 3.73; 95% CrI: 2.02–6.97), anxiety and/or mood disorders (OR = 2.82; 95% CrI: 1.42–5.20), regular physical activity (OR = 2.03; 95% CrI: 1.12–3.66) and healthy dietary behavior (OR = 2.38; 95% CrI: 1.24–4.16) compared with males. In contrast, divorced or widowed participants were less likely to report dissatisfaction with their appearance (OR = 0.25; 95% CrI: 0.08–0.80) and body-dysmorphic concerns (OR = 0.00; 95% CrI: 0.00–0.14) when compared with married respondents. Older respondents showed higher odds of reporting daily smoking (OR = 2.15; 95% CrI: 1.01–4.11) but were also more likely to report regular physical activity (OR = 2.02; 95% CrI: 1.14–3.56), indicating a mixed age-related pattern of health-related behaviours. Overall, these results support that lifestyle and body-image characteristics are associated with socio-demographic determinants (age, sex, and marital status).

### 3.2. Dietary Choices

Dietary choices are shaped by a complex interplay of socio-demographic and psychological factors. We therefore examined how dietary choices in our study population were associated not only with socio-demographic determinants but also with lifestyle and mental health characteristics, including smoking, disordered eating, and self-reported psychological conditions.

**Figure 1:**
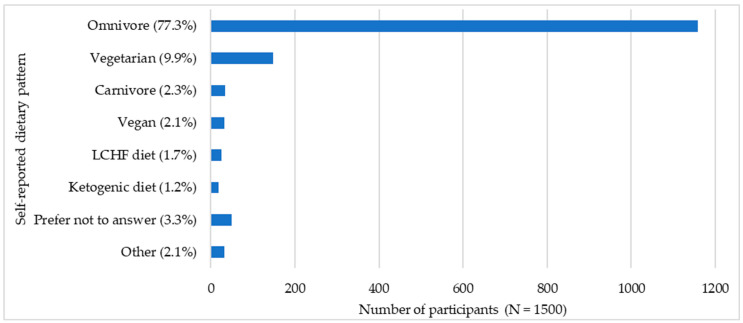
Distribution of self-reported dietary patterns among participants (N = 1500).

As shown in Figure 1, most participants reported following an omnivorous diet, whereas a minority reported alternative dietary patterns (e.g., vegetarian, vegan, carnivore, low-carbohydrate high-fat (LCHF), ketogenic diet) or chose not to disclose. A small subset also reported medically prescribed diets. These findings underscore the predominance of omnivores in the study population while highlighting that a non-negligible proportion of respondents engage in alternative dietary patterns. Given the potential links between such dietary choices and socio-demographic as well as lifestyle characteristics, further analyses were performed to investigate these associations in greater detail.

In subsequent analyses, we focused predominantly on differences between omnivores and vegetarians; due to the small sample sizes of the latter groups, vegetarians and vegans were combined in most analyses to ensure adequate statistical power, although they were treated separately in cases where this was analytically appropriate. This focus was further justified by the results of the statistical analysis, which indicated that vegetarians and vegans differed most markedly from omnivores in their perceptions of PBMDAs. In contrast, other dietary patterns did not exhibit statistically significant differences. For clarity, when referring to the combined vegetarian and vegan group throughout the manuscript, we use the term “vegetarians,” except in instances where vegetarians and vegans are explicitly discussed separately, which is indicated accordingly.

Bayesian multinomial logistic regression was used to identify socio-demographic and lifestyle predictors of dietary patterns. Sex, education, and marital status emerged as the main predictors of vegetarian diet compared with omnivory in the Bayesian analysis (Table 4). Frequentist multinomial logistic regression results were consistent in direction and are provided for context [45,46].

**Figure 2:**
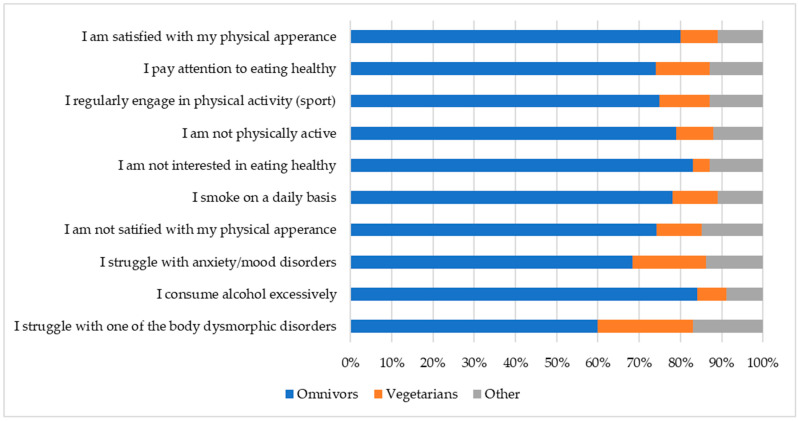
Distribution of lifestyle and self-reported mental health status by dietary pattern among study participants (N = 1500).

Furthermore, we used Bayesian analysis to examine whether dietary pattern was associated with lifestyle choices and self-reported mental health status (Figure 2). Omnivores represented the majority across all categories, whereas vegetarians showed relatively higher proportions in categories related to anxiety and body dysmorphic concerns. The “other” group comprised a smaller share overall but was slightly more represented in the body dysmorphic concerns category (Figure 2).

For context, Pearson’s chi-squared test also indicated evidence of an association between dietary group and response distribution (χ^2^ = 45.66, *p* = 3.33 × 10^−4^); however, the effect size was small (Cramer’s V = 0.06). Although the association was statistically significant, the small effect size suggests that the practical magnitude of the differences between dietary groups is limited, likely reflecting the large sample size (N = 1500) rather than a substantively meaningful association.

Bayesian analysis further revealed that vegetarians had significantly higher odds of reporting psychological difficulties compared with omnivores. Specifically, the odds of reporting body dysmorphic concerns were more than five times greater (posterior OR = 5.19, 95% CrI: 1.45–21.62), while the odds of reporting anxiety were more than three times greater (posterior OR = 3.61, 95% CrI: 1.34–11.51). As the 95% credible intervals did not include 1, these findings suggest a possible association between vegetarian dietary choice and elevated risks of self-reported anxiety and body dysmorphic concerns. However, due to the cross-sectional nature of this study, further research is needed to better understand the direction and mechanisms of this relationship.

### 3.3. Perception and Attitude Towards PBMDAs

**Figure 3:**
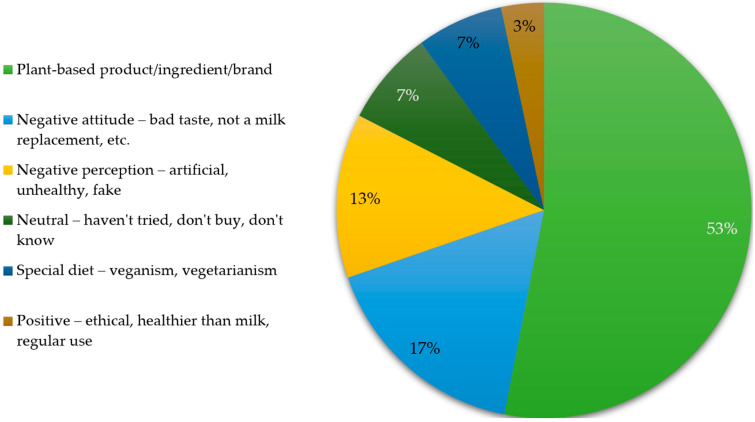
Distribution of associations with PBMDAs (N = 961).

Among participants who reported familiarity with PBMDAs (N = 961), the distribution of general associations with PBMDAs is shown in Figure 3. Overall, neutral associations predominated, whereas explicitly positive associations were uncommon.

**Figure 4:**
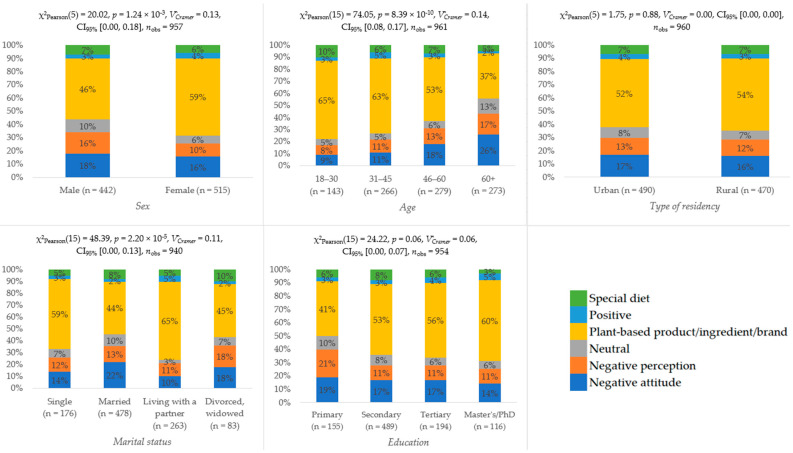
Distribution of attitudes toward and perception of PBMDAs across sociodemographic groups (N = 961).

Furthermore, we investigated how socio-demographic factors influence attitudes toward PBMDAs. The results are shown in Figure 4. Age was the main demographic factor associated with attitudes toward PBMDAs: younger respondents more often linked them to plant origin and specific dietary needs, whereas older respondents (56–60 and 60+) did so less frequently. Marital status showed a smaller association, with married and divorced/widowed individuals more likely to connect PBMDAs to special diets. Sex, education, and residential area were not significantly related to PBMDA attitudes.

Beyond general attitudes, we also explored participants’ beliefs and perceptions regarding milk, dairy products, and PBMDAs. Respondents were presented with 11 statements and asked to indicate their level of agreement on a five-point scale (1 = strongly disagree, 5 = strongly agree). The statements addressed a broad range of issues, including the health effects of replacing dairy with PBMDAs, potential safety concerns such as hormones or antibiotics in milk, and the nutritional importance of dairy in a balanced diet. Further items assessed beliefs about the nutrient composition and calcium fortification of PBMDAs, perceptions of microbiological risks, as well as processing, the use of additives, and the environmental impact of PBMDAs versus dairy products. Statistically significant differences emerged across several socio-demographic groups in relation to perceptions of PBMDAs.

#### 3.3.1. Dietary Preferences

Dietary preference was strongly associated with beliefs about both dairy products and PBMDAs (*p* < 0.001). Overall, vegetarians reported more favourable perceptions of PBMDAs and greater concerns about dairy, whereas omnivores attributed higher nutritional value to dairy. For example, vegetarians agreed more strongly that replacing dairy with PBMDAs has positive health effects (3.44 ± 1.31 vs. 2.57 ± 1.36 in omnivores) and that PBMDAs have a nutrient composition comparable to dairy (3.27 ± 1.26 vs. 2.69 ± 1.23). Concerns about the presence of prohibited substances in dairy products were most pronounced among vegetarians (3.27 ± 1.37), while omnivores expressed the lowest level of concern (2.84 ± 1.37, *p* < 0.001). In contrast, omnivores more strongly endorsed dairy as nutritionally important, including agreement that milk contains essential nutrients (3.94 ± 1.10 vs. 3.41 ± 1.16 among vegetarians) and is an important part of a balanced diet (3.88 ± 1.17 vs. 3.28 ± 1.23; both *p* < 0.001). Omnivores were also more likely to perceive PBMDAs as containing more additives (3.52 ± 1.19 vs. 2.98 ± 1.21) and being more processed (3.62 ± 1.27 vs. 3.30 ± 1.18; all *p* < 0.001), whereas vegetarians more strongly agreed that PBMDAs have a lower carbon footprint (3.44 ± 1.29 vs. 2.64 ± 1.32; *p* < 0.001) and perceived dairy to pose greater microbiological risk (3.14 ± 1.23 vs. 2.59 ± 1.26; *p* < 0.001).

#### 3.3.2. Sex

Sex was associated with a limited subset of perceptions. Compared with males, females more strongly agreed that replacing dairy with PBMDAs has beneficial health effects (2.88 ± 1.44 vs. 2.57 ± 1.36, *p* < 0.001), expressed greater concern about prohibited substances in milk (3.05 ± 1.44 vs. 2.79 ± 1.34, *p* < 0.001), and placed higher importance on choosing calcium-fortified PBMDAs (3.56 ± 1.23 vs. 3.27 ± 1.22, *p* < 0.001). Males rated PBMDAs as containing more additives slightly higher than females (3.48 ± 1.21 vs. 3.31 ± 1.23, *p* = 0.021).

#### 3.3.3. Age

Age was associated with several perceptions of dairy products and PBMDAs. Younger participants reported more favourable views of replacing dairy with PBMDAs, whereas older participants—especially those aged 60+—were less favourable (health benefits: 18–30, 2.87 ± 1.42; 31–45, 3.02 ± 1.39 vs. 60+, 2.48 ± 1.38; *p* < 0.001) and were less likely to consider PBMDAs nutritionally comparable to dairy (3.02 ± 1.28 in 18–30 vs. 2.63 ± 1.25 in 60+; *p* = 0.010). In contrast, the 60+ group attributed greater nutritional importance to dairy (milk contains essential nutrients: *p* = 0.036; balanced diet: highest in 60+ at 3.90 ± 1.15 vs. 3.59 ± 1.28 in 18–30; *p* = 0.004) and perceived PBMDAs as containing more additives (3.54 ± 1.22 vs. 3.29 ± 1.22; *p* = 0.031). Younger participants expressed higher concerns about microbiological risks from dairy (2.92 ± 1.42 in 18–30 vs. 2.56 ± 1.25 in 60+; *p* = 0.008), while perceptions of processing and environmental impact did not differ by age.

#### 3.3.4. Marital Status

Marital status was associated with several perceptions of PBMDAs and dairy products. Participants living with a partner reported the most favourable views on the health benefits of replacing dairy with PBMDAs (3.00 ± 1.41), compared with married (2.59 ± 1.42) and single participants (2.72 ± 1.37; *p* = 0.001). Perceptions of dairy as an important part of a balanced diet also differed by marital status (*p* = 0.004), with higher agreement among married (3.84 ± 1.21) and widowed/divorced participants (3.89 ± 1.17) than among those living with a partner (3.57 ± 1.29). Singles reported greater concern about microbiological risks of dairy (2.87 ± 1.34) than married (2.61 ± 1.28) and widowed/divorced participants (2.57 ± 1.31; *p* = 0.039). Beliefs about the environmental impact of PBMDAs also varied (*p* < 0.001), with participants living with a partner reporting higher agreement that PBMDAs have a lower carbon footprint (2.99 ± 1.35) than married participants (2.62 ± 1.37). No other marital-status differences were statistically significant.

### 3.4. Perceptions of Nutritional Adequacy of PBMDA and Dairy Products

In the final section of the questionnaire, we examined whether dietary preference influenced the perception of nutritional adequacy of different food products.

In the first part of the final question, the study participants were presented with the nutritional declaration of two items, where product A represented a PBMDA for mozzarella cheese and product B represented a mozzarella cheese. They were then asked to indicate which product they perceived as healthier. Importantly, respondents were not informed whether the products were of plant or animal origin. Based on only the nutritional declaration provided, the majority of respondents considered product A to be less healthy than product B. This pattern was consistent and did not vary significantly according to dietary preference (*p* < 0.05).

In a subsequent step, participants were shown the list of ingredients of both products, from which it was now evident to the study participants that product A represented PBMDA, while product B was of animal origin. They were then asked again to evaluate which product they perceived as healthier. Overall, significant differences were observed across dietary groups (*p* < 0.001). The majority of participants considered product A to be less healthy than product B (44.5%), with this perception being most pronounced among omnivores (47.3%). A further 33.7% of respondents reported that product A was “much less healthy” compared with product B, most frequently within the “other” dietary group (40.2%). Only a small proportion of respondents perceived product A as healthier: 3.8% considered it healthier, and 4.5% much healthier than product B. However, this perception was more common among vegans, of whom 21.4% rated product A as healthier and 17.9% as much healthier. Similarly, 8.2% of participants in the “other” group and 8.0% of vegetarians judged product A as much healthier. Finally, 13.5% of respondents stated that there was no difference in healthiness between the two products. This neutral perception was most often reported by vegans (35.7%).

## 4. Discussion

This study provides insights into Slovenian consumers’ habits and perceptions regarding PBMDAs, highlighting differences between vegetarians and omnivores and examining how socio-demographic factors shape these perceptions. Importantly, evidence of this type remains limited in Europe, particularly from smaller Central and Eastern European markets, despite rapid category growth and increasing public interest in plant-based foods. Recent pan-European data from the Smart Protein Project indicate substantial growth in plant-based consumption and rising consumer awareness across EU countries, although adoption remains uneven between regions and demographic groups [2]. Similarly, Good Food Institute Europe reports that over 50% of Europeans report reducing meat intake, yet barriers such as taste, price, and perceived processing continue to constrain broader uptake of plant-based products [3]. In this context, generating nationally representative data on PBMDAs perceptions is therefore relevant not only for academic understanding of consumer segmentation, but also for informing nutrition communication strategies and the evidence base for food-policy decisions in emerging markets such as Slovenia. Our study provides nationally stratified evidence from a Central European market and pinpoints the specific belief dimensions (nutritional adequacy, processing/additives, and sustainability) that structure PBMDAs acceptance and misconceptions.

Consistent with previous research, our findings demonstrate that sex and education were the strongest socio-demographic predictors of vegetarianism and higher PBMDAs acceptance. Comparable sex differences in PBMDA attitudes and consumption patterns have been reported across diverse settings, with females typically expressing more favourable perceptions and stronger health and environmental motivations [27,29,30]. Qualitative research from Poland, Germany, and France similarly found that women more frequently frame PBMDAs within health, ethical, and sustainability narratives, whereas men more often emphasize taste, satiety, and tradition [4]. These patterns are consistent with the broader observation that plant-based alternatives tend to be adopted earlier by consumer groups already engaged with health and sustainability discourses.

The positive association between higher educational attainment and vegetarianism also agrees with previous findings. Prior work suggests that greater nutrition literacy and sustainability awareness—often correlated with educational attainment—are associated with higher openness to PBMDAs and greater perceived healthfulness [14,47]. In contrast, the negative association between marriage or cohabitation and vegetarian or vegan diets has been documented in other contexts. This pattern has been linked to shared household preferences and meal norms that can stabilise traditional omnivorous routines [47]. This suggests that PBMDA adoption may be shaped not only by individual attitudes but also by household-level dynamics, which is relevant when designing dietary guidance or consumer education targeting families.

Beyond socio-demographic determinants, our findings highlight a noteworthy psychological dimension. Vegetarian and vegan participants had higher odds of reporting anxiety and body dysmorphic concerns compared with omnivores. However, the corresponding association at the contingency-table level showed a small effect size (Cramer’s V = 0.06), suggesting limited practical magnitude despite statistical significance—likely influenced by the large sample size. Because these outcomes were measured using self-reported, non-clinical indicators and the study design was cross-sectional, we cannot infer the direction of the associations. The findings may reflect reverse causation (individuals with pre-existing anxiety/body concerns being more likely to adopt vegetarian or vegan diets) or differences in reporting styles (greater symptom awareness or willingness to report among vegetarians). Therefore, these results should be interpreted with caution, as the cross-sectional design precludes causal inference. Existing literature regarding this topic remains mixed. A systematic review by Iguacel et al. [41] reported inconsistent associations between vegetarian diets and mental health outcomes, with substantial heterogeneity across studies. Similarly, Askari et al. [42] found modest associations between vegetarian diets and depressive or anxiety symptoms, but emphasized methodological limitations and potential residual confounding. More recently, Walsh et al. [43] observed that diet quality—rather than vegetarian status per se—was more strongly associated with depressive symptoms, suggesting that poorly planned vegetarian diets may drive adverse associations rather than plant-based eating itself. Thus, our findings may reflect reverse causation (individuals with pre-existing anxiety or body concerns adopting vegetarian diets), reporting differences, or broader lifestyle clustering. Longitudinal studies using validated, clinically anchored measures—such as the approach recommended by Dakanalis et al. (2022) [40] for emotional eating and mental health—are needed to clarify whether these associations reflect causal pathways, reverse causation, or other temporal relationships. While plant-based diets are frequently motivated by ethical or health considerations, these findings underscore the need for healthcare professionals to remain aware of potential overlaps between restrictive eating motives, body dissatisfaction, and vegetarian or vegan dietary identity.

Marked perceptual differences between vegetarians and omnivores also emerged. Vegetarians perceived PBMDAs as healthier, more environmentally sustainable, and safer than dairy products, whereas omnivores expressed greater trust in the nutritional adequacy of dairy and viewed PBMDAs as more processed and additive-rich. Similar perception gaps—often reflecting value-based or ideological evaluation rather than objective composition—have been documented in European consumer studies [15,16,26].

The perception of PBMDAs as “ultra-processed” warrants particular attention. Drewnowski [19] demonstrated that many plant-based milk alternatives meet criteria for ultra-processed classification, yet the nutritional heterogeneity within this category is substantial. Some products are fortified and nutritionally comparable to dairy in selected nutrients, while others are low in protein and micronutrients. Comparative compositional analyses confirm this variability. Walther et al. [23] and Harmer [24] reported that most PBMDAs contain substantially less protein than cow’s milk, unless soy-based or specifically fortified. Jeske et al. [12] showed wide variation in glycaemic and physicochemical properties across products. From a nutritional standpoint, dairy products remain important contributors to calcium, high-quality protein, iodine, vitamin B12, and other micronutrients in European diets [21,22]. Recent reviews [11,13] emphasize that while PBMDAs may contribute to dietary diversification and environmental sustainability goals, their adequacy as direct dairy substitutes depends heavily on fortification and formulation.

Interestingly, in our study, both vegetarians and omnivores tended to rate dairy products more favourably when presented with anonymised nutritional information, suggesting that product category labels and prior beliefs may function as heuristics that shape evaluation independently of composition, an issue also noted in comparative nutritional analyses by McClements & McClements (2023) [18]. It also aligns with research by Cardello et al. [32], who demonstrated that emotional and cognitive associations strongly influence acceptance of plant-based alternatives, sometimes more than objective sensory or nutritional properties. The implication for public health communication is that improving consumer understanding may require not only more information, but clearer and more interpretable information at the point of choice—particularly regarding protein quality, calcium bioavailability, and fortification with vitamins B12 and D.

Age-related differences further highlighted generational divides. Younger adults in our sample expressed more positive views regarding the health and sustainability attributes of PBMDAs, mirroring findings from Martínez-Padilla et al. (2023) [27] and Su et al. (2023) [30]. Younger consumers are more frequently exposed to plant-based messaging, social media influence, and evolving dietary norms. Pan-European market data similarly indicate that younger consumers are leading adoption trends, while older consumers remain more sceptical and concerned about processing and nutritional adequacy [2,3]. In Slovenia, this generational gradient suggests that PBMDA-related knowledge and acceptance may diffuse unevenly across age groups, reinforcing the need for targeted communication strategies that address older consumers’ specific concerns.

Overall, our results indicate that PBMDA acceptance in Slovenia is primarily driven by younger, female, and more educated consumers—consistent with trends reported across Europe and Asia [27,28,29,30,31]. Nonetheless, misconceptions remain widespread, particularly regarding the nutritional equivalence of PBMDAs and dairy. As emphasised by Pointke & Pawelzik (2022) [14] and Clegg et al. (2021) [15], consumers frequently overestimate PBMDA healthfulness and underestimate potential nutrient gaps such as protein quality, calcium bioavailability, and the need for vitamin B12 and D fortification. At the same time, some consumers appear to apply “processing” as a broad negative heuristic, which can obscure meaningful differences in formulation and nutritional profile. In particular, they may not reliably distinguish between relatively simple, fortified PBMDAs (e.g., unsweetened products designed to improve micronutrient adequacy) and variants that are more heavily reformulated, such as sweetened or additive-rich products—thereby reinforcing overly generalised processing-related concerns rather than product-specific evaluation [48,49,50].

These findings have direct implications for Slovenian nutrition policy and consumer education. National dietary guidance should explicitly distinguish between fortified and non-fortified PBMDAs and clarify what constitutes an appropriate nutritional substitute for dairy (e.g., adequate protein and calcium, and—when relevant—B12 and vitamin D). In practice, this could be operationalised through clearer national dietary guidance on PBMDA substitution, targeted public communication for less receptive segments (particularly older consumers), and improved on-pack communication that makes fortification and key nutrients easy to identify. Finally, manufacturers could use these insights to reformulate products (e.g., improving protein/fortification profiles and reducing unnecessary additives) and to communicate these attributes transparently, aligning product development with consumer expectations and nutritional needs. From a public health perspective, balanced communication highlighting both the benefits and limitations of PBMDAs is needed. In parallel, the food industry could improve PBMDA formulation—particularly regarding fortification and additive reduction—to better match consumer expectations.

### Limitations, Strengths, and Future Research

This study has several limitations that should be considered when interpreting the findings. First, the cross-sectional design prevents inference about causality or the directionality of the observed associations between dietary pattern, lifestyle characteristics, and self-reported mental health indicators; observed differences may reflect reverse causation or unmeasured third factors. Second, key constructs—including psychological indicators—were assessed using self-reported, non-clinical measures, which are inherently vulnerable to recall error and social desirability bias. In particular, self-reported mental health symptoms may be influenced by reporting style, differential self-monitoring, or heightened health awareness among individuals choosing vegetarian diets, and may also reflect self-selection effects (e.g., individuals with pre-existing concerns being more likely to adopt specific dietary patterns). Third, although the overall sample was large and stratified, the vegetarian and vegan subgroup was comparatively small, reducing precision for some estimates and necessitating pooling of vegetarians and vegans in most analyses; this limits dietary-pattern specificity and may attenuate subgroup differences. Fourth, PBMDA-related perceptions and open-ended associations were captured via survey responses and analysed descriptively, which may not fully reflect semantic nuance; moreover, the study did not include sensory testing or objective nutritional profiling of PBMDAs and dairy products, limiting conclusions about how perceptions align with product characteristics.

Future research should address these limitations through longitudinal designs to clarify temporal ordering and strengthen causal interpretation. A larger sample of vegetarians and vegans would enable more granular comparisons across dietary subtypes. Psychological outcomes should be assessed using validated screening instruments and, where feasible, clinically anchored measures, ideally triangulated with objective or behavioural indicators. To connect perceptions with product reality, future studies should incorporate objective nutritional assessment (e.g., label-based nutrient profiling and/or biomarker-informed nutrient status where relevant) and sensory evaluation (e.g., blinded tasting and structured sensory profiling), alongside experimental manipulations of product information (e.g., ingredient disclosure and sustainability claims) to test how framing shapes attitudes. In addition, replication of this research design across other European countries would substantially strengthen the evidence base. Although dietary cultures differ, nutrition labelling standards, food information regulation such as Regulation (EU) No 1169/2011 [6], and strategic policy frameworks such as the EU Farm to Fork Strategy [51] create a largely harmonised regulatory environment across Member States. Conducting comparable nationally stratified studies would enable meaningful cross-country comparisons within this shared policy context, helping to distinguish culturally specific perception patterns from those shaped by common regulatory and market structures. Such coordinated research could provide valuable support for both national nutrition communication strategies and EU-level food policy development.

Notwithstanding limitations, this study has several strengths. Data were collected from a large adult sample with stratified recruitment to reflect key demographic distributions, providing robust and broadly generalisable insight into PBMDA perceptions in Slovenia. The integration of socio-demographics, dietary orientation, and psychological indicators offers a multidimensional perspective that is rarely available in nationally structured datasets from Central Europe. In addition, the combination of frequentist tests for descriptive group comparisons with Bayesian multinomial modelling supports more stable inference in the presence of sparse cells and allows probabilistic interpretation of associations. Collectively, these strengths provide a valuable evidence base to inform national nutrition communication, consumer education, and product development and regulatory discussions around plant-based substitutes.

## 5. Conclusions

This study provides a nationally representative overview of PBMDA-related consumption habits and perceptions in adult consumers in Slovenia. It shows clear segmentation by socio-demographic characteristics and dietary patterns. The findings also suggest an association between dietary pattern and self-reported psychological indicators; however, given the cross-sectional design and non-clinical self-report measures, these results should be interpreted with caution. Overall, the study highlights that food choices are shaped not only by nutrition beliefs but also by identity-related preferences and heuristic judgments, underscoring the need for clearer guidance on nutritionally appropriate substitution and for longitudinal research to clarify whether these associations reflect causal effects or pre-existing differences between groups.

## Figures and Tables

**Table 1:** Socio-demographic characteristics of the study participants (N = 1500).

		Responses	Percentage (%)
Sex	Female	747	49.8
	Male	745	49.7
	Other	4	0.3
	Prefer not to answer	4	0.3
Age (years)	18–30	215	14.3
	31–45	375	25.0
	46–60	420	28.0
	60+	490	32.7
Education	Primary	262	17.5
	Secondary (vocational)	297	19.8
	Secondary (technical/general)	493	32.9
	Short-cycle tertiary	180	12.0
	Tertiary (Bachelor/Master)	201	13.4
	Master’s degree (pre-bologna programme)/Doctoral degree	53	3.5
	Prefer not to answer	14	0.9
Marital status	Single	264	17.6
	Married	683	45.5
	In a relationship	388	25.9
	Divorced/widow	129	8.6
	Prefer not to answer	36	2.4
Type of residency	Rural	726	48.4
	Urban	765	51.0
	Prefer not to say	9	0.6
BMI (kg/m^2^)	<18	9	0.6
	18–24.9	488	32.5
	25–29.9	489	32.6
	>30	298	19.9
	Prefer not to say	216	14.4

**Table 2:** Self-reported lifestyle and body image characteristics of participants (N = 1500).

Answer	Responses	Percentage (%)
I am satisfied with my physical appearance	959	64
I pay attention to eating healthy	794	53
I regularly engage in physical activity (sport)	603	40
I am not physically active	319	21
I am not interested in eating healthy	301	17
I smoke daily	250	17
I am not satisfied with my physical appearance	219	15
I struggle with anxiety and/or mood disorders	175	12
I consume alcohol excessively	61	4
I struggle with one of the body dysmorphic disorders	30	2

**Table 3:** Association between socio-demographic characteristics, lifestyle, and body-image indicators.

Predictor	OR *	95% CrI *
Body dysmorphic concerns, divorced/widowed	0.00	0.00–0.14
Dissatisfied with appearance, female	3.74	2.02–6.97
Dissatisfied with appearance, divorced/widowed	0.25	0.08–0.80
Satisfied with appearance, female	1.89	1.09–3.37
Occasional physical activity, female	2.66	1.46–4.94
Regular physical activity, female	2.01	1.11–3.59
Anxiety/mood disorder, female	2.80	1.51–5.39
Smoking daily, older age	2.15	1.00–4.51
Healthy eating, female	2.38	1.35–4.31

* OR = odds ratio; CrI = credible interval; values < 1 indicate decreased likelihood and values > 1 indicate increased likelihood compared with the reference category.

**Table 4:** Comparison of multinomial logistic regression and Bayesian analysis results for predictors of vegetarian diet versus omnivore.

Predictor	Classical OR (95% CI), *p*	Bayesian OR (95% CrI)	Interpretation
Sex	1.55 (1.06–2.27), *p* = 0.024	1.56 (1.07–2.29)	Women had ~55% higher odds of being vegetarian compared with men
Age	ns (all *p* > 0.05)	ns (all CrI include 1)	Age was not a significant predictor
Education	2.32 (1.00–5.37), *p* = 0.050	2.41 (1.01–5.54)	Higher education was associated with >2× higher odds of vegetarianism
Married	0.47 (0.28–0.78), *p* = 0.003	0.46 (0.28–0.77)	Married participants had ~55% lower odds of vegetarianism compared with singles
Living with a partner	0.48 (0.28–0.81), *p* = 0.006	0.48 (0.28–0.81)	Participants who are living with a partner had ~53% lower odds of vegetarianism compared with singles

ns = not significant; *p* ≤ 0.05 = statistically significant; OR = odds ratio; CI = confidence interval; CrI = credible interval.

## Data Availability

The original contributions presented in this study are included in the article. Further inquiries can be directed to the corresponding author.
